# The lateral center-edge angle as radiographic selection criteria for periacetabular osteotomy for developmental dysplasia of the hip in patients aged above 13 years

**DOI:** 10.1186/s12891-020-03515-8

**Published:** 2020-07-25

**Authors:** Daguang Zhang, Xin Pan, Hong Zhang, Dianzhong Luo, Hui Cheng, Kai Xiao

**Affiliations:** 1 Department of Orthopedics, the First Hospital of Jilin University, No.71, Xinmin Street, Changchun, 130021 China; 2 Department of Ophthalmology, the Second Hospital of Jilin University, Changchun, 130041 China; 3 Department of Orthopedics, the 4th medical Center of PLA General Hospital, Beijing, 100048 China

**Keywords:** Developmental dysplasia of the hip, Radiography, Lateral center-edge angle, Periacetabular osteotomy, Tönnis OA classification

## Abstract

**Background:**

This retrospective study sought to delineate the radiographic characteristics of DDH patients over 13 years of age and investigate whether the lateral center-edge angle (LCEA) could serve as radiographic selection criteria for periacetabular osteotomy.

**Methods:**

We enrolled patients with Hartofilakidis type I DDH without dislocation who underwent periacetabular osteotomy between August 2009 and August 2012. LCEA, anterior central edge angle (ACEA), femoral neck-shaft angle (FNSA), Shenton line and Tönnis acetabular index (AI) were evaluated by anteroposterior and 65° false⁃profile pelvic X-ray radiographs in the standing position. Femoral neck anteversion angle (FNA), labral lesion, labral inversion and cartilage lesion were evaluated by direct magnetic resonance arthrography. DDH was categorized by LCEA into four grades (grade I: 10° ≤ LCEA< 20°, grade II: 0° ≤ LCEA< 10°, grade III: -10° ≤ LCEA< 0°, grade IV: LCEA<-10) and osteoarthritis (OA) severity was assessed using Tönnis OA classification. Pearson correlation analysis was done between LCEA and other variables.

**Results:**

Totally patients (274 hips) with a mean age of 27.3 years (range 13–47 years) were included. The mean LCEA was 3.5° (range: − 30° to 20°). Based on LCEA grades, grade I DDH was present in 104 hips, grade II in 40 hips, grade III in 76 hips, and grade IV in 54 hips. Based on Tönnis OA classification, 54.5% hips (150/274) were grade 0, 33.1% hips (91/274) grade 1, 8.4% hips (23/274) grade 2 and 4% hips (11/274) grade 3. Pearson correlation analysis showed a negative correlation between LCEA grade and Tönnis OA grades (*r* = 0.3987; *P* < 0.001). Cochran-Armitage trend test further showed a positive correlation between LCEA grades and labral lesion (*P* < 0.001) and interrupted Shenton line (*P* < 0.001).

**Conclusion:**

The LCEA classification scheme offers a simple and practical approach to categorize the level of acetabulum coverage on the femoral head, hip deformity and characteristics of DDH. Our findings could provide clinically useful guidance for orthopedic surgeons in preparation for periacetabular osteotomy in DDH patients aged above 13 years.

## Background

Developmental dysplasia of the hip (DDH) is frequently seen in pediatric patients with neuromuscular diseases. In many cases, DDH is related with a congenital or developmental deformity or misalignment of the hip joint [1] and cerebral palsy [2]. Anatomically, DDH is characterized by a shallow acetabulum, insufficient coverage and lateral and anterior dislocation of the femoral head [3]. Insufficient coverage of the femoral head and abnormal stress conduction exert chronic stress on the edge of the hip, leading to gradual cartilage lesion and labral tear and, eventually osteoarthritis (OA) [4]. DDH patients are usually treated conservatively or surgically, depending on factors including etiology, severity of deformity, and patient age. Patients aged younger than 13 years usually receive pediatric orthopedic surgery, such as Salter or Dega osteotomy [5]. The specific osteotomy corrects a deformity and improves hip coverage and delays OA onset [6]. For patients aged over 13-years, Bernese periacetabular osteotomy is an effective technique for surgical correction of a severely dysplastic acetabulum [7].

Preoperative radiographic assessment is very critical for Bernese periacetabular osteotomy. Plain X-ray radiography is the most common examination for evaluation of the severity of hip subluxation. Several reference markers are obtained by X-ray to assess DDH development and provide treatment guidance and predict prognosis. The markers include the lateral center-edge angle (LCEA), anterior central edge angle (ACEA), femoral neck-shaft angle (FNSA), femoral neck anteversion angle (FNA), Shenton line and Tönnis acetabular index (AI) [8]. In clinical practice, these markers are used to evaluate the severity of joint subluxation. However, it is unclear which marker correlates with disease severity and could guide surgical operation for patients aged over 13 years with DDH.

In this retrospective study, we sought to delineate the radiographic characteristics of 188 DDH patients (274 hips) over 13 years of age by roentography and direct magnetic resonance arthrography and investigate whether LCEA could serve as radiographic selection criteria for periacetabular osteotomy for DDH patients by studying the correlation between LCEA and OA severity and other radiographic markers. We found that LCEA has very important clinical significance, as it is not only associated with other radiographic markers, but is also correlated with disease onset and severity of OA, suggesting that LCEA could be used as radiographic selection criteria for periacetabular osteotomy for DDH patients aged above 13 years.

## Patients and methods

### Patients

This retrospective study enrolled patients who underwent periacetabular osteotomy at the Department of Articular Surgery, First Affiliated Hospital of People’s Liberation Army (PLA) General Hospital (Beijing, China) from August 2009 to August 2012. Main inclusion criteria were: 1) patients aged at least 13 years with Y-shaped cartilage closed with femoral head epiphysis [9]; 2) LCEA less than 20° and AI more than 10°; 3) Hartofilakidis type I hip dysplasia without dislocation [10]. Main exclusion criteria were: 1) a history of infection or trauma of the hip joint; 2) patients with osteoporosis; 3) post Perthes flat hip deformity; 4) history of femoral shortening osteotomy or of epiphyseal arrest surgery; 5) hip articular or knee joint deformity.

The study was approved by the Institutional Review Board of PLA General Hospital and all patients or their legal surrogates signed informed consent forms for surgery; informed consent of the study was not required because of the retrospective nature of the study. Patient data were anonymized in the paper.

### Radiographic examination

**Fig. 1:**
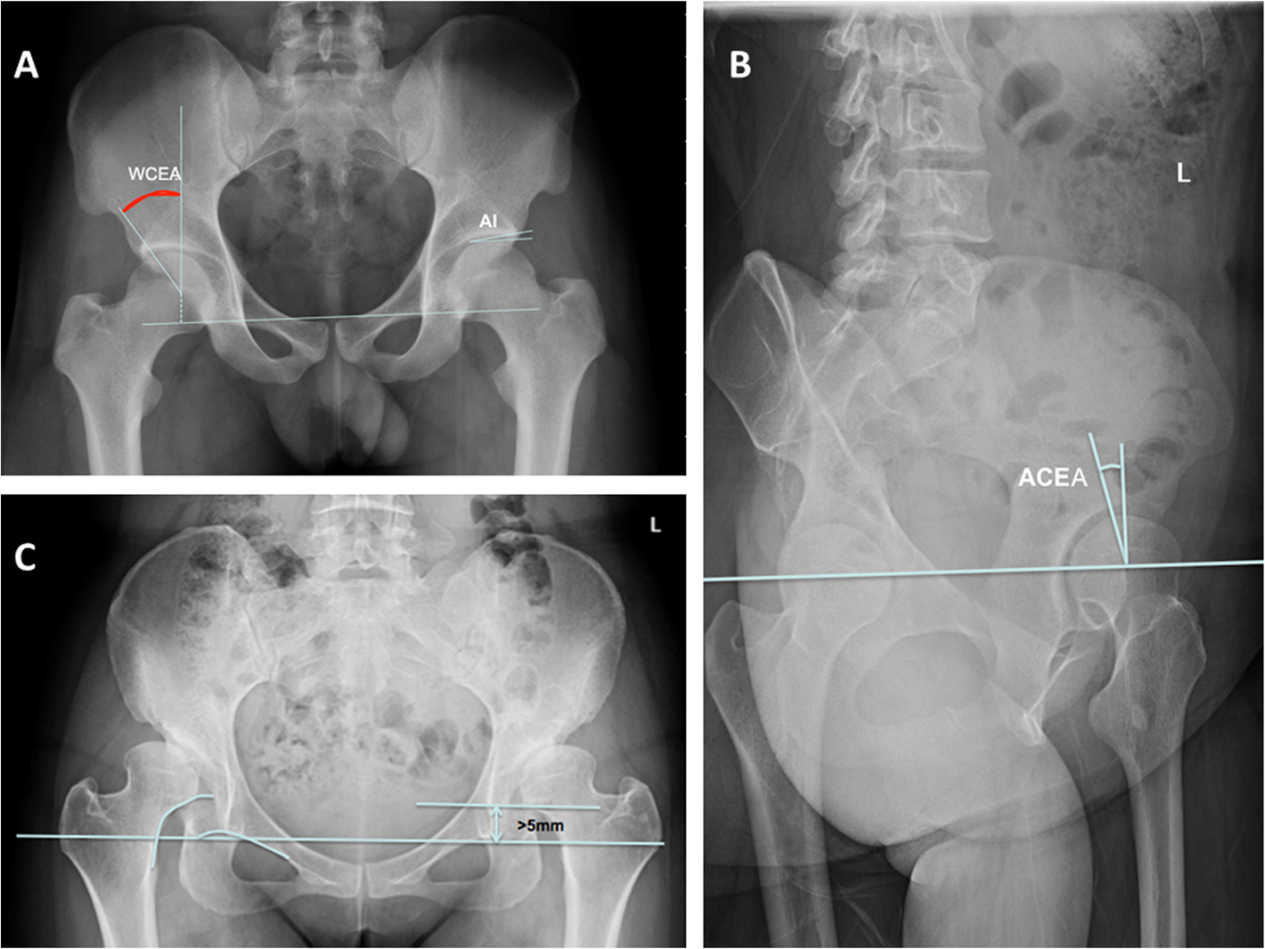
Measurements of radiographic parameters in anteroposterior and 65° false⁃profile pelvic X-ray radiographs in the standing position. **a** Lateral center-edge angle (LCEA) is the intersection angle between the vertical center line of the femur and the lateral edge of the acetabulum; AI is the intersection angle formed with the connection line between the acetabular endocoxa and acetabular outer margin and the pelvic horizontal line. **b** Anterior central edge angle (ACEA) measurement on 65° obliquity on the pelvis of X-ray image. ACEA is the intersection angle formed with the vertical center line of the bilateral femoral head and the anterior margin of the acetabulum. **c** Discontinuity of Shenton line at the pelvis AP position on the X-ray image is defined as a more than 5 mm distance away from the connection line of the obturator superior border vertex and the head and neck junction of the femoral neck

Anteroposterior (AP) and 65° false⁃profile pelvic X-ray radiographs were taken in the standing position with a Definium 600 DR radiography system (General Electric, USA). The reference markers were measured as previously reported [11]. Briefly, 1) LCEA: the angle between the perpendicular line of the center of the femoral head and the lateral edge of the acetabulum in the AP pelvis images, as shown in Fig. 1a. DDH was categorized according to LCEA: grade I: 10° ≤ LCEA< 20°, grade II: 0° ≤ LCEA< 10°, grade III: -10° ≤ LCEA< 0°, and grade IV: LCEA<-10°). 2) ACEA: the perpendicular line of the center of the femoral head and the lateral edge of the acetabulum in the lateral pelvis images, as shown in Fig. 1b. 3) AI: the angle between the inner edge of the acetabulum with the outer edge of the acetabulum extending line and the horizontal line of the pelvis in AP position, as shown as Fig. 1c**.** 4) FNSA: the angle between the femoral shaft axis and the femoral neck axis in the AP position.

Tönnis classification of OA [12] is based on the observation of pelvic X-ray images. Grade 0: hardening of the acetabulum, thickening of the white line of the acetabulum and a normal intraarticular gap. Grade 1: increase in the bone density of the femoral head and acetabulum, and a narrow intraarticular gap (20% narrower than normal). Grade 2: minor pathological changes of femoral joint capsule, and 20–50% narrower of intraarticular gap. Grade 3: major pathological changes of the joint capsule, the intraarticular gap is reduced over 50%, or there is serious femoral deformity or necrosis of the femoral head.

### Direct magnetic resonance arthrography

**Fig. 2:**
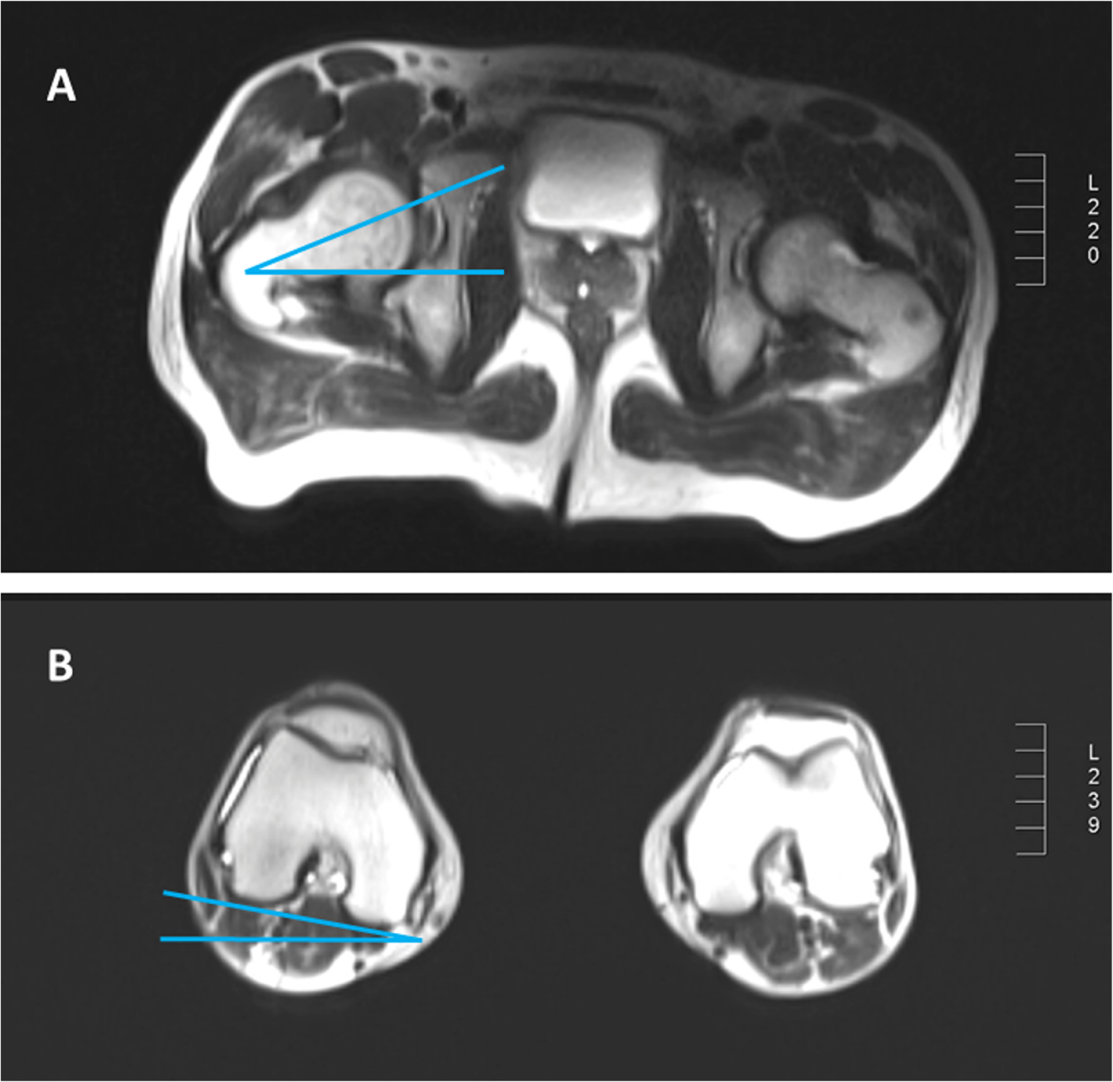
Direct magnetic resonance arthrography of a 30-year-old woman with right developmental dysplasia of the hip. The image shows the measurement and calculation of the femoral neck anteversion (FNA) angle. The cross section of the knee joint and the cross section of the femoral neck are sequentially measured. The axial femoral plane angle is added or subtracted with the intersection angle of the hypocondyle angle and the horizontal line of the knee joint. Angle’s addition is for knee internal flip and angle’s subtraction is for the knee external flip. **a** cross section image of the hip; **b** cross section image of the knee

**Fig. 3:**
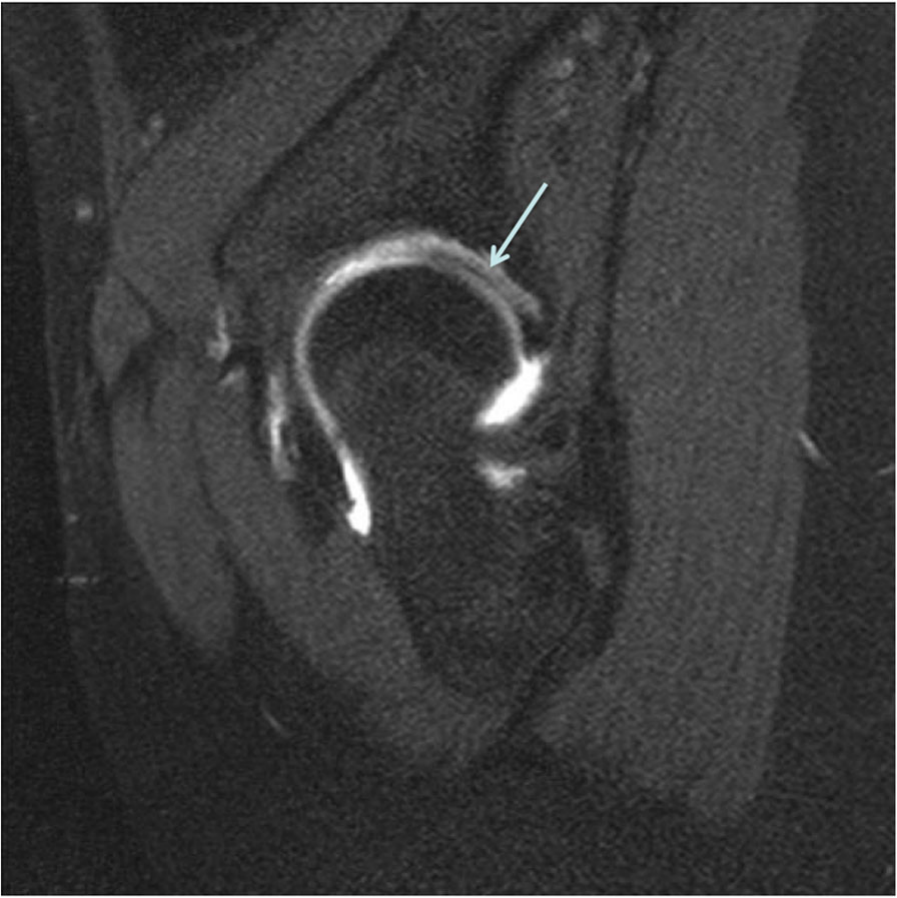
Direct magnetic resonance arthrography of a 31-year-old woman with left developmental dysplasia of the hip. The T1WI MRI image reveals a labral inversion, shown as a stripe of low signal between articular cartilages indicated with an arrow

Patients received an intra-articular injection of 10–15 mL gadolinium solution (gadopentetic acid, dimeglumine, Magnevist 2 mmol/L (Bayer, Leverkusen Germany)) under fluoroscopic imaging with subsequent 1.5 T MR Arthrography (Siemens Avanto, Erlangen, Germany) using a dedicated large flex surface coil at the coronal, sagittal, and transverse sections. The spin echo (SE) sequence was adopted using the small angle with multi-averaging technique. The FNA (Fig. 2), labral lesion, labral inversion (Fig. 3) and cartilage lesion were evaluated by an experienced orthopedic surgeon musculoskeletal radiologist who was blinded to patient data.

### Statistical analysis

Data were analyzed using SPSS Software Version 18.0 (IBM Corp., Armonk NY). Categorical data including gender, Shenton line, labral lesion and Tönnis grade of OA were expressed as number and frequency. Chi-squared test was used for comparison of two groups in labral lesion, labral inversion and Shenton line discontinuity and rank sum test was used for comparison of Tönnis grades of OA of two groups. Quantitative data (age at onset, AI, ACEA, FNA and FNSA) were expressed as mean and standard deviation and comparison between two groups was done using unpaired Student’s *t*-tests, paired *t*-tests, and one-way or two-way ANOVA, where appropriate, and ANOVA was used for multiple groups with Bonferroni test as post-hoc test. Pearson analysis was made correlation between LCEA and age at onset, AI, ACEA, FNA or FNSA and between LCEA and Tönnis grades of OA. Statistical significance was two-sided and was achieved when at *P* < 0.05.

## Result

### Patient demographic and baseline characteristics

**Fig. 4:**
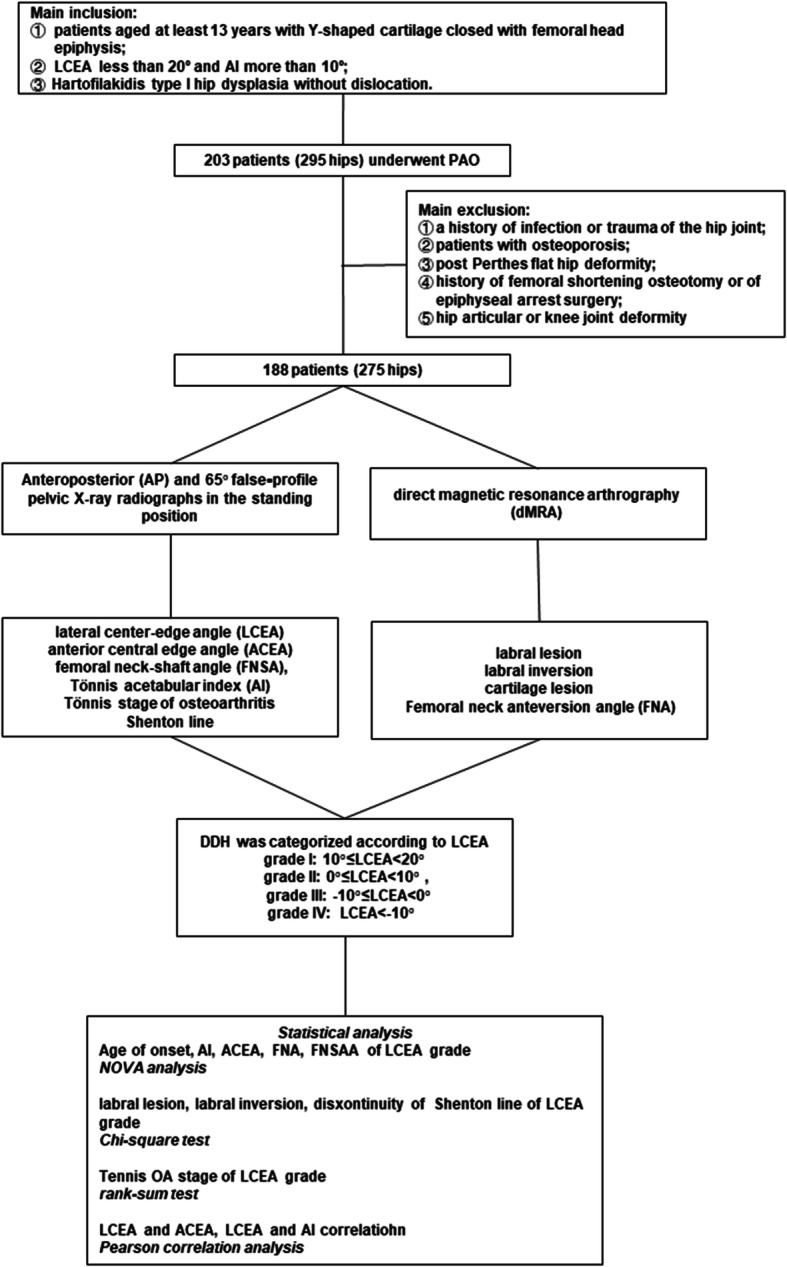
The study flowchart

The study flowchart is shown in Fig. 4. Totally 203 patients (295 hips) who underwent periacetabular osteotomy at our hospital during the study period were screened for eligibility for this retrospective study. Fifteen patients (21 hips) were excluded. Finally, 188 patients (274 hips) were included in this study, and the majority of the population (85.1%) were women. Their mean age was 27.3 years (range 13–47 years). There were 136 left hips and 138 right hips. In addition, bilateral hip dysplasia was found in 53.1% women versus 7% women.

### Radiographic characteristics of the study population

All the hips were evaluated by AP and 65° false⁃profile pelvic X-ray radiographs in the standing position, and the physiological angles of the hip joint, including LCEA, ACEA, and AI, were calculated. The mean LCEA was 3.5° (range: − 30° to 20°; normal range: 25° to 40°). The LCEA ranged between 11° to 20° (grade I DDH) in 104 hips, between 1° to 10° (grade II DDH) in 40 hips, from − 9° to 0° (grade III DDH) in 76 hips, and below − 10° (grade IV DDH) in 54 hips.

According to Tönnis classification, 54.5% hips (150/274) were grade 0, 33.1% hips (91/274) grade 1, 8.4% hips (23/274) grade 2 and 4% hips (11/274) grade 3. In addition, discontinued Shenton line was found in 30.2% (83/274) hips, acetabular labral damages in 29.8% (82/274) hips, acetabular labral inversion in 5.1% (14/274) hips, and cartilage damage in 14.9% (14/274) hips.

### Correlation between LCEA and AI and ACEA

**Fig. 5:**
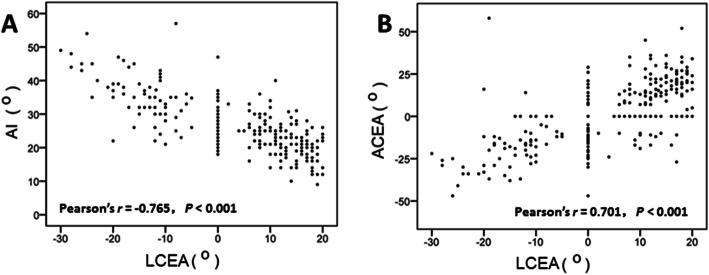
The correlation of LCEA with Shenton line and Tönnis acetabular index (AI) is shown in (**a)**; the correlation of LCEA with ACEA is shown in (**b**)

The mean age of onset was 30.9 ± 7.6 years in grade I DDH patients, 28.8 ± 7.7 years in grade II DDH patients, 25.3 ± 8.8 years in grade III DDH patients, and 23.1 ± 8.3 years in grade IV DDH patients, showing that younger patients had more advanced DDH (*P* < 0.01) **(**Table 1**)**. In addition, patients with different LCEA grades differed significantly in AI; patients with higher LCEA grade had significantly higher AI (*P* < 0.001). Our Pearson correlation analysis showed a negative correlation between LCEA and AI (*r* = − 0.765; *P* < 0.01) (Fig. 5a). Furthermore, ACEA progressively decreased as LCEA grade increased: ACEA was 14.8° ± 13.4° in grade I, 2.6° ± 12.1° in grade II, − 5.9° ± 13.4° in grade III, − 18.9° ± 16.5° in grade IV. Pearson correlation analysis revealed a significant positive correlation between LCEA and ACEA (Fig. 5b). We found no statistically significant difference in FNSA or FNA among patients with different LCEA grades (*P* > 0.05).

**Table 1:** Age of onset, AI, ACE, FNSA and FNA stratified by LCEA classification^d^

Variable	Classification of LCEA				*P*
	Grade I	Grade II	Grade III	Grade IV	
No.	104	40	76	54	
Age of onset, years	30.9 ± 7.6	28.8 ± 7.7	25.3 ± 8.8^a^	23.1 ± 8.3^ab^	< 0.001
AI °	20.4 ± 4.9	25.5 ± 5.1^a^	29.2 ± 5.8^ab^	36.5 ± 7.3^ab^	< 0.001
ACEA°	14.8 ± 13.4	2.6 ± 12.1^a^	−5.9 ± 13.4^ab^	−18 ± 16.5^abc^	< 0.001
FNA °	24.9 ± 10.7	26.0 ± 14.0	27.7 ± 12.4	29.0 ± 13.4	0.190
FNSA °	132.9 ± 7.6	134.1 ± 7.5	132.6 ± 6.3	135.0 ± 7.6	0.234

ACEA: anterior central edge angle; AI: Tönnis acetabular index; LCEA: Lateral center-edge angle; FNA: femoral neck anteversion angle; FNSA: femoral neck-shaft angle

^a^*P* < 0.05 versus grade I; ^b^*P* < 0.05 versus grade II; ^c^*P* < 0.05 versus grade III

^d^Data are expressed as mean ± SD unless otherwise specified

### Correlation between LCEA and OA severity

As hip dysplasia is associated with OA, we examined if LCEA correlated with OA and cartilage damage. As shown in Table 2, most patients with LCEA grade I and II had Tönnis grade 0 OA while most LCEA grade III and IV patients had Tönnis grade 1 OA. Spearman correlation analysis showed a negative correlation between LCEA grade and severity of OA (*r* = 0.3987; *P* < 0.001) and a correlation efficient of 0.387(95%CI, 0.281–0.492) between LCEA grade and Tönnis OA classification (*P* < 0.001). As acetabular labrum damage is often seen in hip dysplasia and OA, next we analyzed if LCEA was associated with labral lesion and labral inversion. Labral lesion was found in 18.3% (19/104) joints in LCEA grade I patients, 20% (8/40) joints in LCEA grade II patients, 36.8% (28/76) joints in LCEA grade III patients, and 50% (27/54) joints in LCEA grade IV patients. There was a positive association between LCEA and labral lesion (Cochran-Armitage trend test, *P* < 0.001). In addition, a significantly greater proportion of LCEA grade IV patients with labral inversion were seen versus LCEA grade I patients (*P* < 0.01).

**Table 2:** Tönnis osteoarthritis classification, labral lesion, labral inversion and discontinuity of Shenton line stratified by LCEA grades, [n (%)]

Variables	Classification of LCEA				*P*	*P*
	Grade I	Grade II	Grade III	Grade IV		
Tönnis osteoarthritis classification					< 0.001^c^	–
Grade 0	81 (77.9)	21 (52.5)	31 (40.8)	16 (29.6)		
Grade I	18 (17.3)	14 (35.0)	34 (44.7)	25 (46.3)		
Grade II	3 (2.9)	3 (7.5)	7 (9.2)	10 (18.5)		
Grade III	2 (1.9)	2 (5.0)	4 (5.3)	3 (5.6)		
Labral lesion	19 (18.3)	8 (20.0)	28 (36.8)	27 (50.0)	0.001^a^	< 0.001^b^
Labral inversion	1 (1.0)	1 (2.5)	5 (6.6)	8 (14.8)	0.003^a^	< 0.001^b^
Discontinuity of Shenton line	6 (5.8)	8 (20.0)	29 (38.2)	40 (74.1)	< 0.001^a^	< 0.001^b^

^a^chi-square test or Fisher test, ^b^Cochran-Armitage trend test; ^c^Spearman classification correlation parameter 0.387(95%CI, 0.281–0.492)

Shenton line is an imaginary curved line drawn along the inferior border of the superior pubic ramus (superior border of the obturator foramen) and along the inferomedial border of the neck of femur. This line should be continuous and smooth in normal condition. Shenton line is interrupted in hip dysplasia and other deformities. We found that there was a positive correlation between LCEA grade and interrupted Shenton line (Cochran-Armitage trend test, *P* < 0.001). Only 5.8% (6/104) LCEA grade I cases had an interrupted Shenton line versus 74.1% (40/54) in LCEA grade IV cases (*P* < 0.001) **(**Table 2**).**

## Discussion

Currently, there is lack of radiographic markers that correlate with severity of hip subluxation and could guide surgical treatment of DDH patients aged over 13 years. In the present study, we established a novel LCEA classification scheme as radiographic selection criteria for periacetabular osteotomy in DDH patients aged above 13 years and found that LCEA negatively correlates with severity of OA. In addition, the study demonstrated that LCEA negatively correlates with AI and positively correlates with ACEA and labral lesion. Our findings could offer clinically useful guidance for orthopedic surgeons in preparation for periacetabular osteotomy in DDH patients aged above 13 years.

DDH is frequently seen in pediatric patients with congenital or developmental deformity or misalignment of the hip joint. Patients with hip dysplasia usually treated conservatively or surgically, depending on the etiology, the severity of deformity, and patient age etc. Patients younger than 13 years of age may receive pediatric orthopedic surgery, such as Salter or Dega osteotomy [5]. For patients over 13 years of age, Bernese periacetabular osteotomy is an effective technique for surgical correction of a severely dysplastic acetabulum [7].

In order to provide better radiographic assessment of hip dysplasia before surgery, we analyzed the following radiographic markers: LCEA, ACEA, FNSA, FNA, AI, and Shenton line [8] in 188 DDH patients (274 hips) by using AP and 65° false⁃profile pelvic X-ray radiographs in the standing position. Among those markers, we found that LCEA is a valuable marker that it is not only associated with other radiographic markers, but also correlated cartilage damage and OA severity. A prior study considered that clinical symptoms and secondary lesions in DDH patients were determined by factors like gender, age, occupation, and degrees of hip strain [13]. We found that women accounted for a much higher proportion of our cohort than men, and more women had bilateral joint involvement than men. Both the left and right hip were equally affected. The mean age of patients in this study was 27.3 years; interestingly, the LCEA classification was correlated with age of onset, as patients with early onset tended to have more severe LCEA grade.

Among all the radiographic markers we measured, LCEA is a very important marker to evaluate disease severity. LCEA has positive association with ACEA and negative correlation with AI, and it is not correlated with FASA and FNA. The normal ACEA is around 50° [14, 15], and a smaller ACEA is associated with more severe hip dysplasia. AI is another useful measurement, which is formed by the junction of Hilgenreiner’s and a line drawn along the acetabular surface. Normally AI should be below 30° [16, 17]. A smaller AI indicates more severe hip dysplasia. We found that LCEA correlate both ACEA and AI, which makes LCEA a better marker to evaluate hip dysplasia.

In addition, our study showed LCEA is associated with joint pathological changes, including cartilage damage, labral lesions, labral inversions and OA severity. Patients with a smaller LCEA tended to have more severe OA. Labral lesions and labral inversions are common intra-articular lesions of DDH [18]. The relationship between intra-articular lesions and the severity of hip deformity has been unclear. Our study showed that 30% DDH patients had a labral lesion and 15% had a labral inversion and illustrated that smaller LCEA values correlated with more severe hip deformity and the presence of labral lesions and labral inversions. Direct magnetic resonance arthrography is necessary before osteotomy to delineate intra-articular deformity and lesion, and it is crucial to deal with the labral lesion and labral inversion as part of osteotomy to guarantee a successful operation. In addition, our study demonstrated that LCEA correlated with Shenton line continuity, which is indicative of the degree of hip dysplasia. The worse lateral and anterior coverage of the acetabulum on the femoral head is related to of hip semi-dislocation, which is consistent with a previous study [19].

Due to the complexity of DDH, classification methods, such as Crowe and Hartofilakidis, have been developed [10, 20]. The Crowe and Hartofilakidis classification methods have significance in terms of guiding artificial articular replacement surgery of late-stage adult DDH OA; however, the two methods are limited for more accurate classification in hip protection treatment. Thus, there is a need for more clinical and practical classification methods [21, 22]. Femoral deformity and abnormal version also contribute to the development of OA in dysplastic hips. In our study, we excluded patients with femoral malformations or deformities and therefore did not evaluate the contribution of femoral deformity and abnormal version to the development of OA in dysplastic hips. It will be of clinical importance to evaluate such patients in future investigations. The current understanding of the etiology of hip OA is that certain variations in hip anatomy such as femur-acetabular congruity predispose the joint to the development of OA [23] and abnormal bony morphology of the femur and/or acetabulum is believed to initiate damage to the articular cartilage and acetabular labrum and may predispose the hip to early OA [24].

In summary, LCEA is an important radiographic parameter and clinical index for DDH patients. It not only correlates with other radiographic markers, but also reflects hip joint damage, and it can provide valuable information for diagnosis and prognosis. Our classification scheme based solely on LCEA could allow the use of less radiation and imaging, therefore potentially saving radiation, time, and cost. However, LCEA does not offer a guide to optimal correction or predict outcomes.

## Conclusion

LCEA is the key parameter among all clinical X-ray radiographic parameters, with clinical significance in terms of guidance of osteotomy, hip protection, evaluation of the level of deformity and estimation of OA grade. The LCEA classification scheme offers a simple and practical approach to categorize the level of acetabulum coverage on the femoral head, hip deformity and the characteristics of DDH.

## Data Availability

The datasets used and/or analysed during the current study are available from the corresponding author on reasonable request.

## Abbreviations

LCEA: Lateral center-edge angle
ACEA: Anterior central edge angle
FNSA: Femoral neck shaft angle
FNA: Femoral neck anteversion angle
AI: Tönnis acetabular index
